# Supplementary material for: Dynamic organization of cerebellar climbing fiber response and synchrony in multiple functional components reduces dimensions for reinforcement learning
Source: eLife. 2023 Sep 15;12:e86340. doi: 10.7554/eLife.86340 (PMC10531405; doi:10.7554/eLife.86340)
Supplement: Supplementary file 1. — (a) The number of PCs sampled in individual AldC compartments at different learning stages. (b) The number of trials for each cue-response condition at different learning stages. [file elife-86340-supp1.docx]

| AldC compartment | 7+ | 6- | 6+ | 5- | 5+ | 5a- | 5a+ | 4- | Sum |
| --- | --- | --- | --- | --- | --- | --- | --- | --- | --- |
| 1st | 74 | 227 | 273 | 218 | 295 | 222 | 104 | 49 | 1462 |
| 2nd | 118 | 333 | 502 | 527 | 383 | 317 | 164 | 61 | 2405 |
| 3rd | 206 | 243 | 279 | 434 | 503 | 444 | 341 | 128 | 2578 |
| Sum | 398 | 803 | 1054 | 1179 | 1181 | 983 | 609 | 238 | 6445 |

| Learning stage | 1st | 2nd | 3rd | Sum |
| --- | --- | --- | --- | --- |
| HIT | 599 | 1357 | 1832 | 3788 |
| FA | 677 | 795 | 285 | 1757 |
| CR | 158 | 543 | 1528 | 2229 |
| MISS | 118 | 36 | 47 | 201 |
| Sum | 1552 | 2731 | 3692 | 7975 |
